# Chronic Rhinitis

**Published:** 1912-07

**Authors:** 


### Chronic Rhinitis.

This is occasionally an infectious disease, but more often due to either autotoxemia, or to some constitutional disease, such as syphilis, rachitis, tuberculosis, etc. The mucous membrane is infiltrated and the connective tissue hyperplastic, and there is a constant secretion of muco-pus. The trouble is recurrent, rather than chronic. It is usually associated with structural changes in the nasal passages, which give no particular trouble until the hypertrophied membrane is aggravated by exposure to cold, or to any of the exciting causes mentioned in the acute form. Then there ensue all the symptoms of the acute form, excepting the severity and acuteness of the onset. The discharge is thicker and more sluggish than in the acute variety.

In the atrophic type (ozena) the membrane and glands shrink and exfoliate, and emit a peculiarly offensive odor.

*Treatment.*—Constitutional measures are of the utmost importance. If possible, secure change of climate to a reasonably dry and constant atmosphere. Temperature is not so important. Give appropriate treatment for the underlying condition, such as syphilis or tuberculosis. In general, syrup of iodide of iron, Fowler's solution, and cod liver oil are indicated. Enjoin plenty of exercise in the fresh air, daily tepid baths and rub-downs, and a plain, simple diet.

Local cleanliness and disinfection is of the highest importance.

All crusts must be removed thoroughly every day, or oftener if necessary, with a warm solution of sodium bicarbonate and common salt, 2 to 5 grains of each to the ounce, and the nasal membranes then thoroughly douched with warm 50 per cent solution of Listerine, after which they should be protected with a spray of liquid albolene.

In the atrophic form the cleansing measures are even more important, since the fetor results from decomposing crusts. Remove all crusts carefully and gently by a constant flow of warm bicarbonate solution, entering at one nostril and flowing out of the other. In obstinate cases add to the solution 3 drams of ammonium chloride and 10 drops of carbolic acid to the pint; or where the fetor is very bad, substitute sodium salicylate for the bicarbonate. Prevent the reformation of the crusts by douching thoroughly with straight Listerine, followed by oil sprays, pinol, eucalyptol, and menthol, with albolene as a base to protect the membranes.

When a reasonably clean surface has been attained, paint the membrane twice daily with the following:

Tr. iodii............................Min. xx
Kali iod..............................gr. v
Glycerini.............................dr. ij
Aquae rosae...........................q. s. oz. j

Or, if there be deep ulcers, touch them with the following:

Aristol ...............................

Ol. ricini...........................aa gr. 150

Collodion............................gr. 1200

—*Selected.*

---

Hemorrhage from an old, indurated gastric ulcer is a much more serious matter than bleeding from a more recent ulcer, since in the former the vessel may be unable to collapse and allow clotting.—*American Journal of Surgery.*

---

Traumatic aneurysm, after temporary clamping of the artery, can often be treated by suture if the surgeon goes about it deliberately, when at first impression the case seemed to demand ligation and obliteration of the vessel.—*American Journal of Surgery.*

